# Associations between ionomic profile and metabolic abnormalities in a murine model of sodium sulfide induced alopecia areata

**DOI:** 10.3389/fphar.2025.1507348

**Published:** 2025-05-14

**Authors:** Luning Li, Zhen Sun, Wenxue Sun, Yujuan Zhai, Na Ding, Wei Wang

**Affiliations:** ^1^ Clinical Medical Laboratory Center, Jining First People’s Hospital, Jining, Shandong, China; ^2^ Department of Clinical Pharmacy, Jining First People’s Hospital, Jining, Shandong, China; ^3^ Department of Clinical & Translational Medicine, Jining First People’s Hospital, Jining, Shandong, China; ^4^ Department of Dermatology, Jining First People’s Hospital, Jining, Shandong, China

**Keywords:** gas chromatography-mass spectrometry, metabolome, inductively coupled plasma mass spectrometry, ionome, alopecia areata, tofacitinib

## Abstract

**Background:**

Alopecia areata (AA) is a common autoimmune disorder marked by non-scarring hair loss, which imposes significant psychosocial stress on patients. To investigate key metabolites and ions involved in AA’s pathogenesis, we utilized gas chromatography-mass spectrometry (GC-MS) for non-targeted metabolomics and inductively coupled plasma mass spectrometry (ICP-MS) for ionomics.

**Methods:**

A total of 36 six-week-old Kunming mice were divided into control (n = 12), an AA model (n = 12), and tofacitinib-treated groups (n = 12). A mouse model of AA was established by sodium sulfide (Na_2_S) induction in both the model and treatment groups, while the treatment group (n = 12) received tofacitinib treatment at a dose of 1 mg/kg. GC-MS was used to determine the metabolic profiling in serum samples, and ICP-MS was applied to assess ionomic changes in the serum samples. Potential metabolites and ions were identified using orthogonal partial least squares-discriminant analysis (OPLS-DA). Subsequently, MetaboAnalyst 5.0 and the Kyoto Encyclopedia of Genes and Genomes database (KEGG) were used to map the metabolic pathways. Spearman correlation analysis was conducted to identify relationships and potential regulatory interactions between differential metabolites and individual ions.

**Results:**

Metabolomics analysis revealed that D-lactic acid, glycolic acid, linoleic acid, petroselinic acid, and stearic acid are key differential metabolites between the control, AA model, and tofacitinib groups. Pathway analysis highlighted that the biosynthesis of unsaturated fatty acids and linoleic acid metabolism are pivotal pathways implicated in the onset and progression of AA. Furthermore, ionomics analysis identified magnesium, aluminum, titanium, and nickel as differential ions among the three groups. The integrated metabolomics and ionomics analysis indicated that linoleic acid, a key differential metabolite according to the KEGG database, shows a positive correlation with phosphorus, vanadium, magnesium, and zinc. Among these, Mg^2+^ (Mg^2+^) play a crucial role in modulating CD8^+^ T cell infiltration, thereby influencing the disease progression in AA.

**Conclusion:**

Tofacitinib inhibits CD8^+^ T cell infiltration in hair follicles affected by sodium sulfide-induced AA by modulating the linoleic acid metabolism-Mg^2+^ pathway. Our findings offer new insights and potential avenues for the clinical diagnosis and treatment of AA, suggesting that targeting metabolic and ionic pathways could enhance therapeutic outcomes.

## 1 Introduction

Alopecia areata (AA) is a common autoimmune condition characterized by non-scarring hair loss, affecting individuals all ages, with a global prevalence of approximately 0.1%–0.2% (Lauren et al., 2017; Dimitra Aikaterini et al., 2022). Although AA can manifest at any age, it is most frequently observed in individuals under 40 and show no significant variations in incidence based on race or gender. Furthermore, it is often associated with other autoimmune diseases (Virginia et al., 2022; Teruki et al., 2023). The exact etiology of AA is still not fully understood; however, it is widely believed to involve a T-cell-mediated immune response, with hair follicles being the target. The onset of AA is typically precipitated by certain factors that disrupt immune privilege in anagen-phase hair follicles, leading to the activation of T lymphocytes and the release of various inflammatory mediators, which ultimately damage to the hair follicles and result in hair loss (Hongwei et al., 2015). In addition, the self-reactive T-cell stimulating factors interleukin-2 (IL-2) and interleukin-15 (IL-15) are implicated in the progression of AA. The serum levels of IL-15 in patients with AA were significantly elevated compared to the control group and showed a positively correlation with the severity of the disease, and the expression of both IL-2 and IL-15 was markedly increased in the affected skin (Ebrahim, Rehab Mohammed et al., 2019). The current treatment of AA presents several significant challenges. Tranditional therapies, including glucocorticoids and immunosuppressants, have shown limited efficacy in managing moderate to severe cases of AA. Moreover, patients who respond to these treatments remain at high risk of recurrence (Ebrahim, Rehab Mohammed et al., 2019). Patients with AA often heightened psychological distress, driven by both hair loss and the lack of effective treatment options, which can lead to anxiety and depression (Annemieke de et al., 2018). Therefore, more attention is needed to develop improved strategies for the prevention and treatment of AA.

In recent years, the rapid advancements in science and technology have led to increasing interest in ionomics and metabolomics. Ionomics, an emerging field following genomics, proteomics, and metabolomics, focuses on the study of the distribution and composition of both metal and nonmetal elements within biological systems. By integrating ionomics with metabolomics, researchers can gain deeper insights into the biological roles and mechanisms of these elements (García-Sevillano et al., 2014; Jean-Phillip et al., 2023). Metabolomics, a key part of systems biology, centers on analysis of an organism’s metabolites (Joanna, 2004), emphasizing their diversity, quantity, and variability. Research has increasingly demonstrated that the gut microbiome and metabolome play a pivotal role in influencing skin health and inflammatory skin conditions, including AA (Fiehn, 2002; Mahmud et al., 2022; Sánchez-Pellicer et al., 2022b). Studies suggest that altering the gut microbiota or metabolites, through interventions such as fecal microbiota transplantation (FMT), can improve the efficacy of immunotherapies. This indicates a promising avenue for developing therapies that target the gut microbiome or metabolome in the treatment of skin-related autoimmune diseases like alopecia areata (Baruch et al., 2021; Sánchez-Pellicer et al., 2022a).

In this study, we established an AA mouse model and administered tofacitinib investigate its effects. Serum samples were collected from three groups: control mice, AA model mice, and tofacitinib-treatment AA mice. These samples were then analyzed using gas chromatography-mass spectrometry (GC-MS) for non-targeted metabolomic and inductively coupled plasma mass spectrometry (ICP-MS) for ionomic analysis. Our findings revealed that linoleic acid and magnesium were key differential metabolites and ions among the groups. AA model mice and tofacitinib-treated AA mice. These results provide valuable insights and offer a novel theoretical framework for future strategies in preventing and treating AA.

## 2 Materials and methods

### 2.1 Chemicals and reagents

Heptadecanoic acid (purity ≥ 98%), chromatographic grade methanol, and pyridine were obtained from Macklin Biochemical Technology Co., LTD (Shanghai, China). O-methoxylamine hydrochloride was sourced from J&K Scientific Co., LTD (Beijing, China). N, O-Bis (trimethylsilyl) trifluoroacetamide with with 1% trimethylchlorosilane, pentobarbital sodium, and picric acid solution were purchased from Sigma-Aldrich (St. Louis, MO, USA). Tofacitinib was obtained from Selleck (Houston, TX, United States). Pure water was purchased from Wahaha Group Co., LTD (Hangzhou, China).

### 2.2 Animal and treatment

#### 2.2.1 Sample size calculation

The segment pertaining to animal experiments was carried out in accordance with the principles of Randomized Controlled Trial (RCT), and the sample size was determined based on a power analysis to ensure sufficient statistical power (80%) to detect a significant difference (p < 0.05) between the two groups. It was calculated that a minimum of 11.2 animals were required in the control group compared to the model group and a minimum of 10.04 animals were required in the model group compared to the dosing group. To ensure robustness, we included 12 animals in each of the three groups. Meanwhile, this sample size ensures reliable statistical analysis while adhering to the 3R principles of ethical animal research.

The calculation process used to determine the minimum number of animals required for this study are as follows:

a) Effect Size (d): Based on similar studies investigating metabolic alterations in tissues using metabolomics, an expected effect size (Cohen’s d) of approximately 1.5 (large effect size) was used.
b) Power (1−β): To ensure sufficient statistical power to detect significant differences between groups, we set the power to 80% (0.8).
c) Significance Level (α): A two-tailed significance level of 0.05 (α = 0.05) was selected.
d) Calculation Method: Using the formula for two independent groups:

n=2×Zα/2 +Zβ 2×σ2/Δ2

$o\;Z\alpha /2=1.96\;\left(\text{for }\alpha =0.05\right)$

$o\;Z\beta =0.84\;\left(\text{for }80\%\text{ power}\right)$

o σ = pooled standard deviation (based on prior studies or pilot data, approximately 39% in the control group compared to the modeled group, and approximately 36% in the modeled and administered groups)

o Δ = minimum detectable difference between the means of the two groups (assumed to be 45%, based on biological relevance)

Substituting these values into the formula:

① Control group compared to model group:

n=2×1.96 +0.84 2×0.39 2/0.45 2

n ≈ 11.2 animals per group.

② Model group compared to tofacitinib group:

n=2×1.96 +0.84 2×0.36 2/0.45 2

n ≈ 10.04 animals per group.

#### 2.2.2 Animal breeding and molding

A total of 36 male KM mice, 6 weeks old with body weights ranging from 30 to 35g, were purchased from Pengyue Laboratory Animal Breeding Co., LTD (Jinan, China).

The mice exhibited no abnormalities in skin or hair. They were housed under controlled conditions: a 12-h light/dark cycle, temperature maintained at 20°C–22°C, humidity at 65%, and had free access to food and water. All mice were provided with a standardized diet, receiving the same standardized chow and water. Food intake was monitored daily to ensure no significant differences between groups. Stratified randomization was performed based on body weight using Research Randomizer (v4.0). Each mouse was assigned a unique ID and then allocated to the control group (n = 12), model group (n = 12), or tofacitinib group (n = 12) *via* block randomization (block size = 6) to ensure balanced weight distribution. After 3 days of adaptive feeding, 24 mice were randomly selected for hair removal. In accordance with established methodologies documented in prior literature (Chen et al., 2019; Li et al., 2022), we employed topical sodium sulfide (Na_2_S) application to establish the murine alopecia areata model. As a reducing agent and principal component of potent depilatory formulations, sodium sulfide achieves chemical depilation through keratin protein dissolution in hair shafts, providing thorough hair removal at low cost, its potent irritancy may cause cutaneous burns and contact dermatitis. Current evidence indicates that CD8^+^ T cells accumulate in perilesional inflammatory tissues, as observed in psoriasis (Ma et al., 2023) and related disorders. Given our research focus on evaluating tofacitinib’s therapeutic efficacy against alopecia areata, we utilized sodium sulfide to chemically simulate inflammatory responses in the scalp microenvironment. Subsequent to the induction of scalp inflammation, CD8^+^T cells are prone to accumulate in the vicinity of the inflamed tissues. This phenomenon bears remarkable resemblance to the pathological process of alopecia areata, thereby such chemical induction enables rapid and streamlined assessment of drug intervention outcomes. The detailed modeling protocol consisted of: A symmetrical 2 cm × 2 cm skin area prepared on the back of each mouse, marked with picric acid as the experimental area. Hair in the experimental area was treated with 8% sodium sulfide alcohol solution, applied with a sterile cotton swab. The success of AA induction was validated based on the following criteria: a) Daily photographic scoring of hair loss area (graded 0–3); b) Inclusion criterion: ≥50% hair loss in the target area by day 14. From the AA model group, 12 mice were randomly administered tofacitinib (1 mg/kg) in normal saline solution for 7 consecutive days. Mice in the control group and the AA group were injected with normal saline over the same period. All animal experiments were conducted in accordance with the guidelines for the use of experimental animals and were approved by the Animal Experiment Ethics Committee of Jining First People’s Hospital (Approval number: JNRM-2024-DW-135).

### 2.3 Blinding procedures

a) Laboratory technicians were blinded to group labels, with solutions coded as A/B/C; b) GC-MS/ICP-MS operators analyzed samples in randomized batches; **c)** Two independent pathologists, blinded to treatment conditions, scored H&E/IF images.

### 2.4 Sample collection

Prior to sampling, mice were fasted for 4 h with free access to water. Serum collection was consistently performed between 09:00 and 10:00 a.m. to minimize circadian metabolic variations. Mice were anesthetized using 1% pentocarbital sodium (50 mg/kg), followed by the collection of blood samples through enucleation (removal of the eyeballs). Serum were separated from the blood samples by centrifugation at 4000 rpm for 10 min at 4°C. The collected serum samples were then stored in at −80°C for future analysis.

### 2.5 Metabolomics

#### 2.5.1 Samples preparation

A total of 100 μL of serum was mixed with 350 μL of a heptadecanoate methanol solution. The mixture was then centrifuged at 14,000rpm at 4°C for 15 min. After centrifugation, the supernatant was transferred into a 1.4 mL Eppendorf tube and dried under nitrogen at 37°C. Subsequently, 80 μL of O-methoxylamine hydrochloride pyridine solution was added, and the mixture was incubated at 70°C for 90 min. Following this, 100 μL of N, O-Bis (trimethylsilyl) trifluoroacetamide with 1% trimethylchlorosilane was added and incubated at 70°C for 1 hour. After thorough mixing, the solution was centrifuged at 3,000 rpm at 4°C for 2 min, followed by filtration through a 0.22 μm microporous filter.

#### 2.5.2 GC-MS analysis

Biofluids were analyzed using GC-MS with an Agilent 7000C mass spectrometer coupled to a 7890B gas chromatography system (Agilent, Santa Clara, CA, USA). The gas chromatography system was equipped with an HP-5MS fused quartz capilary column (30 m × 0.25 mm × 0.25 μm), and helium was employed as the carrier gas at a flow rate of 1 mL/min. A 1 μL aliquot of the sample was injected into the system with a 50:1 split ratio. The injection port temperature was set at 280°C, with the transmission line temperature at 250°C, and the ion source temperature at 230°C. Mass spectrum was performed using electron impact ionization at an energy level of −70 eV. Data acquisition occurred at a frequency of 20 spectra per second, and the full scan mode was employed across a mass-to-charge ratio (m/z) range of 50–800.

#### 2.5.3 Measures to prevent and correct batch effects

a) To reduce batch effects, a pooled quality control (QC) sample was prepared by mixing equal portions from each sample after extraction. During instrumental analysis, a QC sample was analyzed every six samples to monitor metabolic variation trends. Batch effects were then corrected using the metaX R package, which applies a support vector regression (SVR)-based correction method; b) Batch effects were assessed through clustering heatmaps to evaluate data distribution across different batches and experimental conditions, ensuring no significant discrepancies between batches.

#### 2.5.4 Data processing and multivariate analysis

The raw GC-MS data were processed using Agilent Mass Hunter software (versionB.07.00). Data preprocessing includes alignment, retention time correction, baseline filtering, and deconvolution. A sample bank containing all QC samples was constructed, and unknown metabolites in QC samples were identified using the National Institute of Standards and Technology (NIST) 14 GC-MS library. Metabolites with a similarity score of ≥80% were selected for further identification. Subsequently, a broad-spectrum library was established and employed for metabolites matching in experimental samples. Peak areas were normalized using Microsoft Excel™ (Microsoft, Redmond, WA, USA) followed by orthogonal partial least squares discriminant analysis (OPLS-DA) using SIMCA-P 14.1 (Sartorius Stedim Biotech, Goettingen, Germany). Differences between groups were evaluated using two-tailed Student’s t-tests. Metabolites with a variable importance in projection (VIP) score >1.0 and *p* values <0.05 were considered as potential differentially expressed metabolites. MetaboAnalyst 5.0 (http://www.metaboanalyst.ca) was utilized for clustering analysis. Pathway analyses were conducted using the metabolic pathway results from MetaboAnalyst 5.0, integrated with the Kyoto Encyclopedia of Genes and Genomes (KEGG) database (http://www.kegg.jp).

### 2.6 Ionomics

#### 2.6.1 Samples preparation

The constant volume tube was first rinsed with pure water, followed by a wash with 20% nitric acid, and a final rinse with pure water. The tube was then placed in an oven to dry. A 0.7 mL sample was added to a 50 mL constant-volume tube, followed by 1 mL of concentrated nitric acid. The tube cap was secured but loosened slightly to allow pressure release. The sample was then heated on a plate at 130°C for 2 h, during which red-brown fumes were observed. Heating was discontinued once the fumes dissipated, indicating complete digestion, and the sample became clear and transparent. The solution was then cooled to room temperature and diluted with ultrapure water to a final volume of 25 mL.

#### 2.6.2 ICP-MS

ICP-MS analysis of biological fluids was conducted using the NexION 2200 inductively coupled plasma mass spectrometer (PerkinElmer, Shelton, CT, USA). This instrument features Universal Cell Technology (UCT), enabling operation in Standard mode, Collision mode with Kinetic Energy Discrimination (KED), and Reaction mode with dynamic bandpass tuning (DBT). The three reaction gas channels offer flexible solutions to minimize or eliminate spectral interferences. In Collision mode, an inert gas like helium interacts with analytes and interferences. Polyatomic interferences, due to their larger cross-sectional diameters compared to elemental ions of the same mass (e.g., 40Ar35Cl^+^ vs 75As^+^), undergo more collisions, losing kinetic energy and failing to pass through the cell’s energy barrier. In Reaction mode, a reactive gas interacts with either the analyte or the interfering species, shifting the analyte to a new mass (e.g., 75As^+^ reacts with O2 to form 75As16O^+^ at mass 91) or neutralizing the interference (e.g., 35Cl16O^+^ reacts with NH3, forming neutral ClO). The Analyzer Quadrupole then separates the analyte mass, ensuring interference-free detection.

#### 2.6.3 Quantitation of elements

The quantification of element content in samples was calculated using the formula:

$$X=\frac{C\times V_{2}}{V_{1}}$$

*X* represents the concentration of elements in the sample, expressed in nanograms per milliliter (ng/mL).

*C* detectes the detected elements concentration, expressed in micrograms per liter (ug/L).

*V*_*1*_ is the sample size, expressed in milliliters (mL).

*V*_*2*_ refers to the volume of sample in milliliters (mL).

To assess the precision of measurements, the relative deviation (RD) between two parallel samples was calculated using the formula:

$$RD=\frac{\left|X_{1-}\left.X_{2}\right|\right.}{\bar{X}}$$

*RD* is Relative deviation.

*X*_*1*_ and *X*_*2*_ are the element concentrations in two parallel sample (ng/mL).

$\bar{X}$ represents the average concentration of the two parallel measurements.

The relative standard deviation (RSD) was determined using the formula:

$$RSD=\frac{\mathrm{SD}}{\bar{X}}\times 100\%$$

*RSD* is the relative standard deviation.

*SD* represents the standard deviation of the measurements, and $\bar{X}$ is the mean concentration.

This ensures the accuracy and reliability of elemental quantification in the sample analysis.

#### 2.6.4 Quantitative outcome evaluation

To ensure accurate and reliable results, the linear correlation coefficient of the standard curve must be less than 0.995. Additionally, the RD between two independent measurements, conducted under repeatability conditions, should not exceed 20%. Furthermore, the RSD of these independent measurements must be less than 10%. These criteria guarantee the precision and consistency of the quantitative analysis outcomes.

### 2.7 Histology

Skin samples were fixed in formalin, dehydrated, and subsequently embedded in paraffin. Sections were cut to a thickness of 4 μm, then deparaffinized and rehydrated. Hematoxylin and eosin (H&E) staining was performed using reagents from Abcam. Images of the stained sections were captured using an OLYMPUS BX53 microscope, with a scale bar of 100 μm.

### 2.8 Immunofluorescence (IF) staining

Immunofluorescence (IF) staining was performed on formalin-fixed, paraffin-embedded tissue sections. After dewaxing and rehydration, the slides were incubated overnight at 4°C in a humidified chamber with primary antibodies against CD8 (Abcam, ab178089, 1:200). Following thorough washing, the sections were incubated with Alexa Fluor 594-conjugated secondary antibodies (Invitrogen). Finally, all slides were mounted with an antifade reagent containing DAPI (S36938, Thermo Fisher Scientific) to counterstain nuclei. Fluorescent images were captured using a ZEISS LSM880 confocal fluorescence microscope.

### 2.9 Statistical analysis

Statistical analyses of metabolites and ions were performed using SPSS19 (IBM, Armonk, NY, USA). For data that followed a normal distribution, a Student-t test was employed, while non-parametric test were conducted for data that did not meet the assumption of normality. *P* < 0.05 was considered statistically significant.

## 3 Results

### 3.1 Metabolomics multivariate data analysis

**FIGURE 1:**
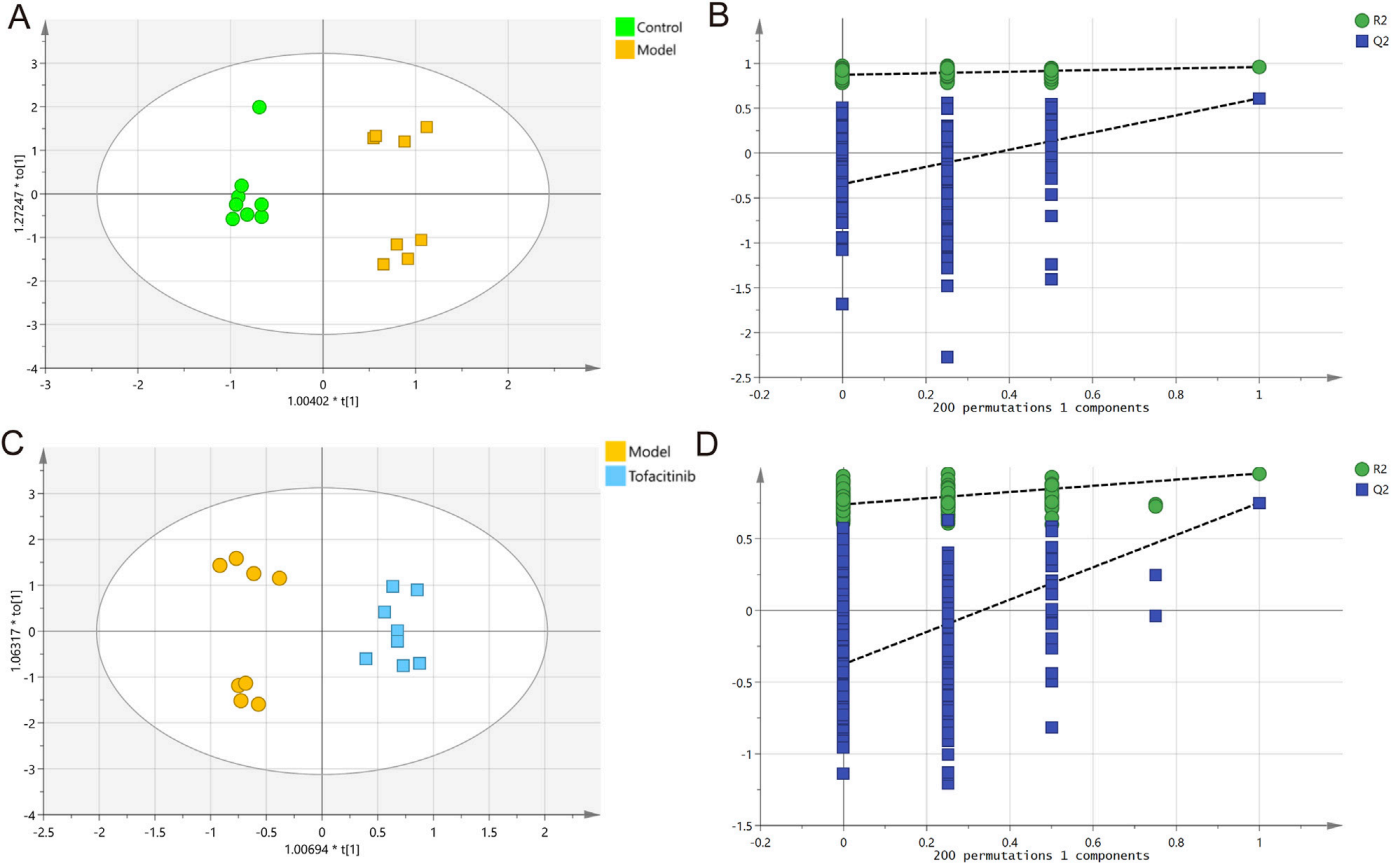
Multivariate statistical analysis of GC-MS data. OPLS-DA score plots **(A)** and 200 permutation tests **(B)** in control and model groups. OPLS-DA score plots **(C)** and 200 permutation tests **(D)** in model and tofacitinib groups.

To investigate the relationships between multiple independent and dependent variables, orthogonal partial least squares discriminant analysis (OPLS-DA) models were employed. The OPLS-DA score plots and validation plots for the comparisons between the control/model groups and the model/tofacitinib groups were constructed (Figures 1A–D). The proportion of variance explained by the OPLS-DA model (R^2^Y) was 96% and 95.5%, respectively. The cumulative Q^2^ values (0.611 and 0.751) were all above 0.50, indicating the model’s robustness and reliability. These results confirm the high quality and reliability of the OPLS-DA model, making it suitable for exploring differences between the control, model, and tofacitinib groups in this study. This analysis further facilitated the identification of key metabolites across the three groups.

### 3.2 Differential metabolites identification

**FIGURE 2:**
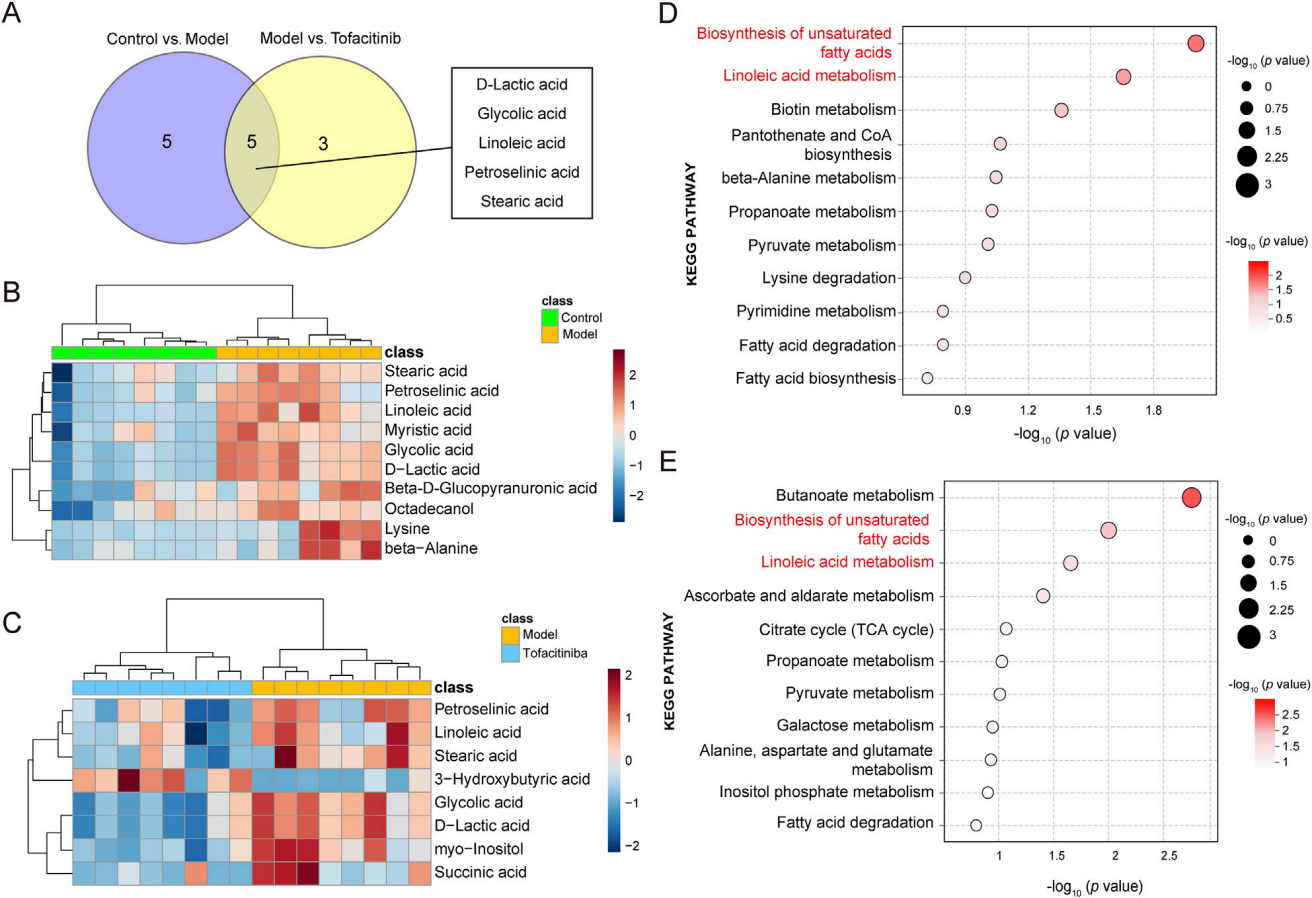
Differential metabolites identification and pathway analysis**. (A)** Venn diagram of the differential metabolites between the model and tofacitinib groups. Heatmap **(B)** and pathway analysis **(D)** of differential metabolites in the model group compared with control group. Heatmap **(C)** and pathway analysis **(E)** of differential metabolites in the tofacitinib group compared with model group.

In the OPLS-DA model, VIP>1 and *p* < 0.05 metabolites were the difference metabolites between the two groups. Based on these criteria, 10 differential metabolites were identified between the control group and the model group, while 8 differential metabolites were found between the model group and the tofacitiniba group. A Venn diagram (Figure 2A) revealed five common metabolites across both comparisons: D-lactic acid, glycolic acid, linoleic acid, petroselinic acid, and stearic acid. Notably, the model group exhibited an upregulation of 10 metabolites, including stearic acid, petroselinic acid, and linoleic acid, compared to the control group (Figure 2B). Furthermore, 3-Hydroxybutyric acid was significant elevated in the tofacitinib group, whereas the remaining metabolites showed a marked increase in the model group (Figure 2C).

### 3.3 Metabolic pathway analysis

To further investigate the metabolic distinctions among the control group, model group, and tofacitinib-treatment group, KEGG pathway analyses were performed on the identified differential metabolites. The results revealed that the differential metabolites between the control and model groups were primarily associated with the biosynthesis of unsaturated fatty acids, linoleic acid metabolism and biotin metabolism. In contrast, the differential metabolites between the model and tofacitinib groups predominantly impacted the butanoate metabolism, biosynthesis of unsaturated fatty acids, and linoleic acid metabolism (Figures 2D, E). These findings suggest that the biosynthesis of unsaturated fatty acids and the linoleic acid metabolism may represent key metabolic pathways contributing to the pathogenesis and progression of AA.

### 3.4 Ionomics multivariate data analysis

**FIGURE 3:**
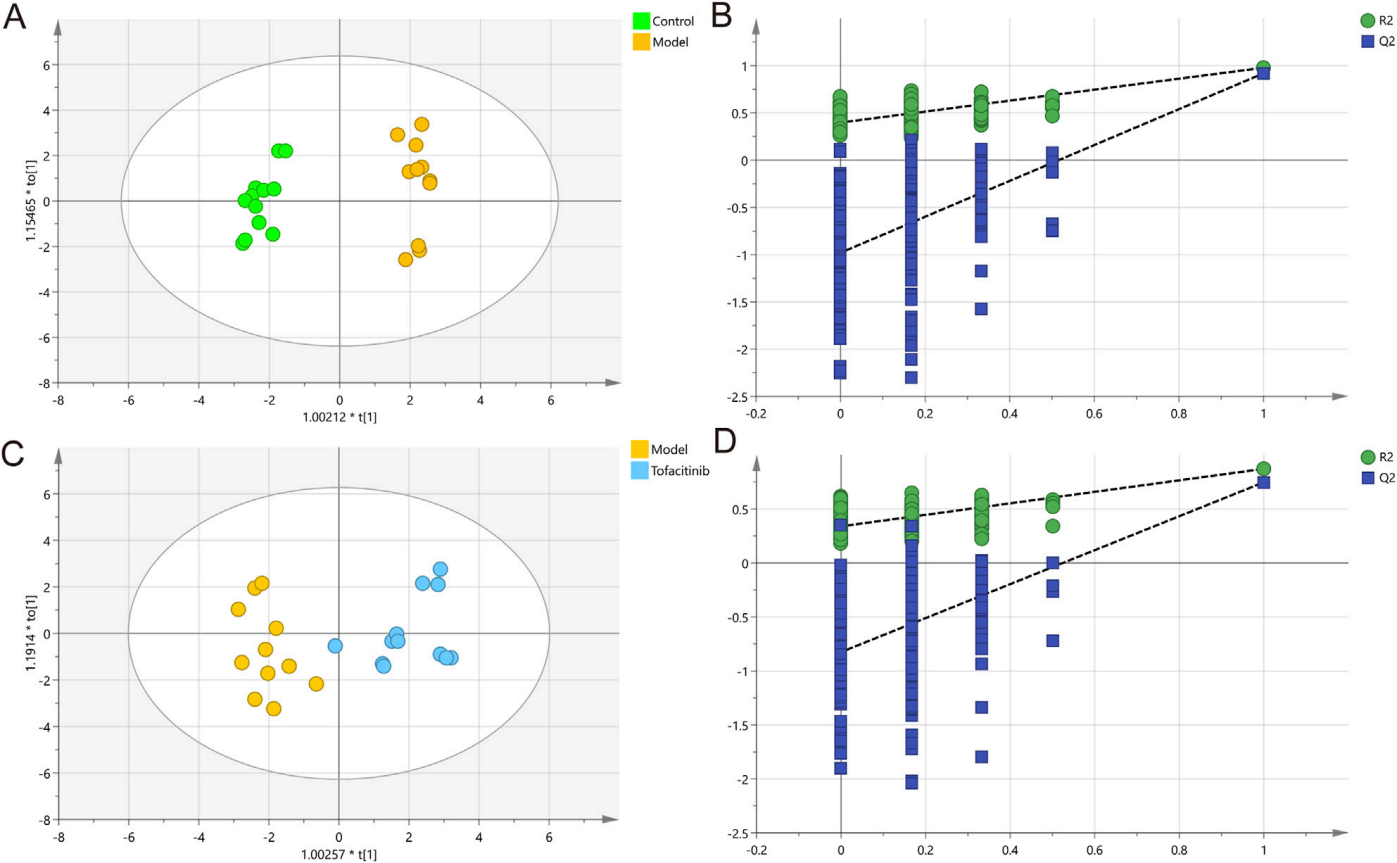
Multivariate statistical analysis of ICP-MS data. OPLS-DA score plots **(A)** and 200 permutation tests **(B)** in control and model groups. OPLS-DA score plots **(C)** and 200 permutation tests **(D)** in model and tofacitinib groups.

Figure 3 presents the results of ionomics OPLS-DA score analysis and permutation testing. The OPLS-DA model was established to distinguish the differential ions among the three groups. This model demonstrated significant separation in ion levels between the control and model groups, as well as between the model and tofacitinib groups (Figures 3A, C **)**. The corresponding permutation test results further confirmed that the OPLS-DA model did not exhibit overfitting (Figures 3B, D). The variance explained by the two OPLS-DA models was 97.8% and 87.2%, respectively. Cumulative Q2 values (0.918, 0.748), both exceeding 0.50, indicate the model’s robustness and reliability. The high quality and reproducibility of this OPLS-DA model confirm its appropriateness for investigating the ionomic differences among the three groups in this study, facilitating the subsequent identification of key ions.

### 3.5 Differential ions identification

**FIGURE 4:**
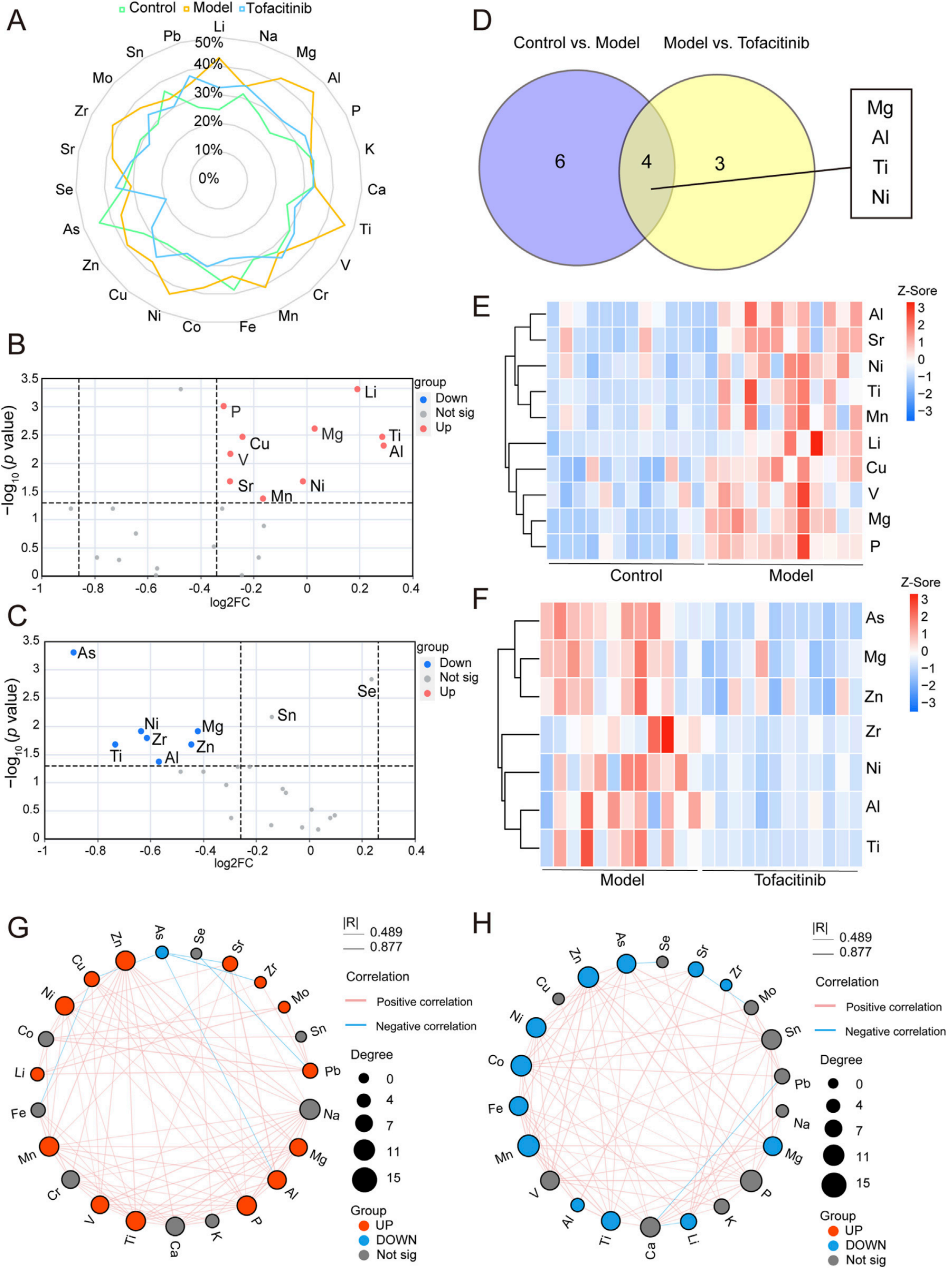
Differential ions identification and correlation analysis**. (A)** The radar chart of ions variation across the control, model, and tofacitinib groups. **(B)** The volcano plot of differential ions between the control and model groups. **(C)** The volcano plot of differential ions between the model and tofacitinib groups. **(D)** Venn diagram of the differential ions between the model and tofacitinib groups. Heatmap **(E)** and correlation analysis **(G)** of differential ions in the model group compared with control group. Heatmap **(F)** and correlation analysis **(H)** of differential ions in the tofacitinib group compared with model group.

In the three groups, 10 upregulated ions were identified between the control and model groups (FC > 1.2, *p* < 0.05), while 7 downregulated ions were found between the model and tofacitinib groups (FC < 0.83, *p* < 0.05). Box plots and ROC curves were employed to depict the differential expression and model accuracy of each ion, respectively (Supplementary Figure S1, S2). A radar chart demonstrates the variation of 23 ions across the control, model, and tofacitinib groups (Figure 4A; Supplementary Figure S3). Volcano plots (Figures 4B, C) visually highlight ions with significant differences, while a Venn diagram reveals that magnesium, aluminum, titanium, and nickel showed significant changes simultaneously in both sets of comparison (Figure 4D). A heatmap further illustrates the differential ion levels among the three groups. Compared to the control group, the model group showed significantly elevated serum levels of lithium, phosphorus, magnesium, copper, titanium, vanadium, aluminum, nickel, strontium, and manganese (Figure 4E). Conversely, in the tofacitinib group, arsenic, nickel, magnesium, zirconium, titanium, zinc, and aluminum were significantly downregulated compared to the model group (Figure 4F). Correlation analysis of serum ion levels (Figures 4G, H) revealed significant positive correlations between magnesium, aluminum, nickel, titanium, and zinc, a key element involved in hair growth. Thus, our findings suggest that magnesium, aluminum, titanium, and nickel may play crucial roles in the progression of AA.

### 3.6 Integrated analysis of metabolomics and ionomics

**FIGURE 5:**
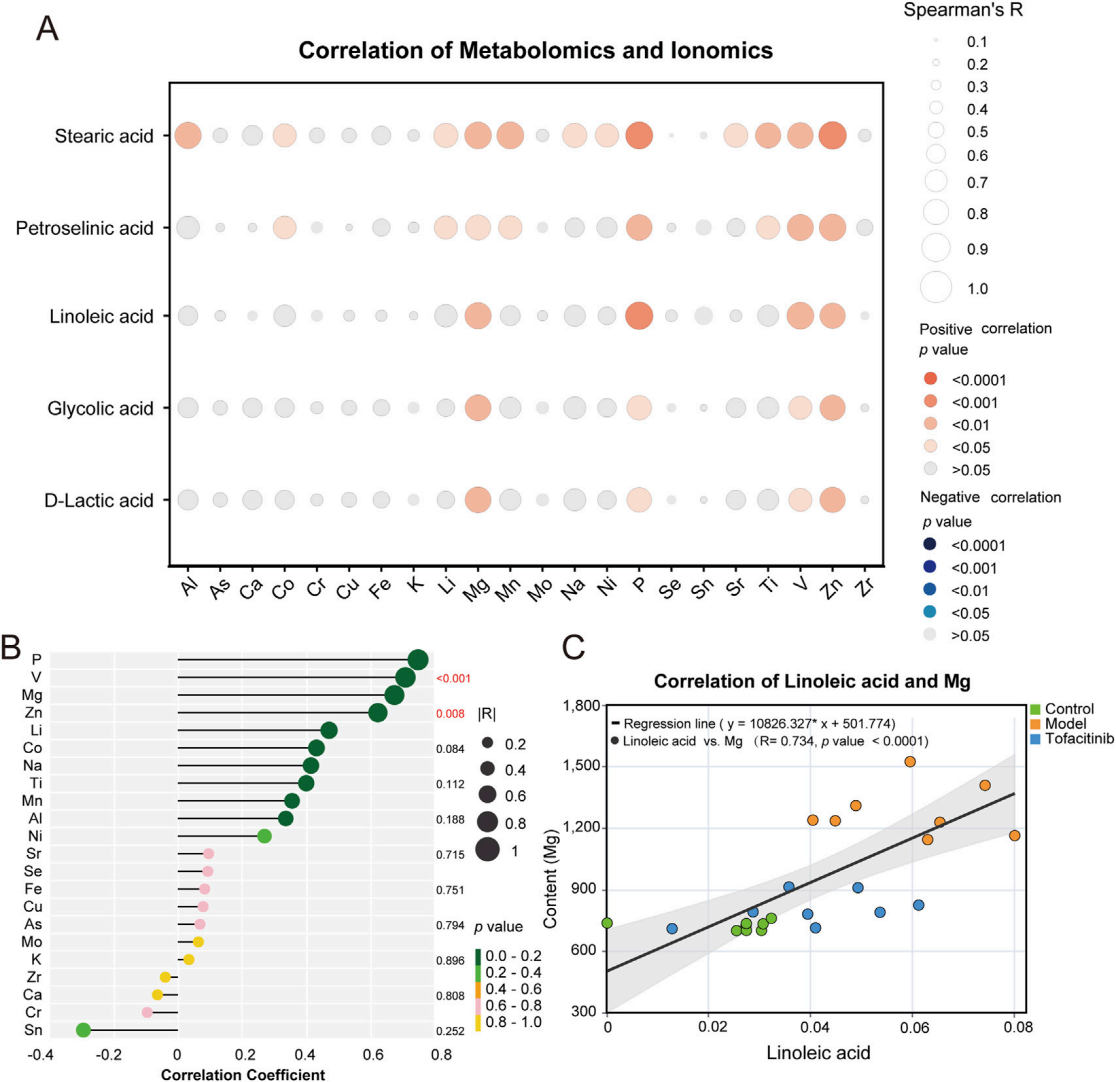
Integrated analysis of metabolomics and ionomics**. (A)** The correlation (Spearman’s R) between five differential metabolites and various ions. **(B)** The lollipop plots of five differential metabolites and various ions. **(C)** The correlation (Spearman’s R) of linoleic acid and magnesium.

To explore the relationship between differential metabolites and ions in AA, we examined the correlation between five differential metabolites identified through metabolomics and various ions. Our analysis revealed a significant positive correlation between these metabolites and both magnesium and zinc (Figure 5A). Notably, linoleic acid, a key differential metabolite identified in the KEGG database, was positively correlated with phosphorus, vanadium, magnesium, and zinc (Figure 5B). Of these ions, magnesium garnered particular attention. Further analysis focused on the correlation between linoleic acid and magnesium, lithium, nickel, aluminum, and zinc in serum samples from the control, model, and tofacitinib-treated groups (Figure 5C; Supplementary Figure S4). The correlation between linoleic acid and magnesium was especially prominent in the progression of alopecia areata, showing a significant positive correlation (R = 0.734, *p* < 0.0001) (Figure 5C).

### 3.7 Histology and immunofluorescence (IF) staining

**FIGURE 6:**
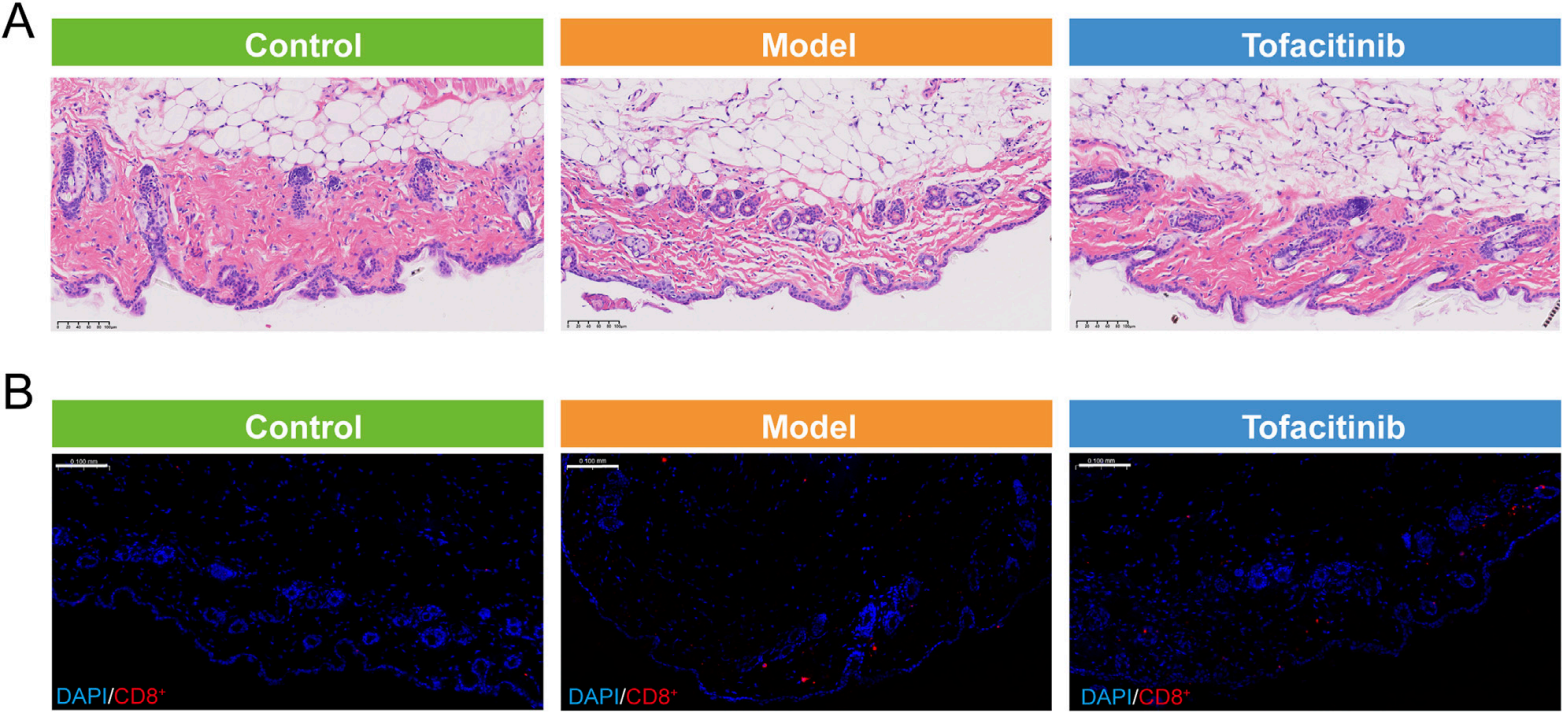
Histology and immunofluorescence (IF) staining**. (A)** The Hematoxylin and eosin (H&E) staining of control, model, and tofacitinib groups. **(B)** The immunofluorescence (IF) staining of CD8^+^T cell in control, model, and tofacitinib groups.

Hematoxylin and eosin (H&E) staining demonstrated that the AA (Model) group, in comparison to the Control group, exhibited a pronounced reduction in skin inflammation capacity, as indicated by significant epidermal thickening and a notable decrease in hair follicle count. Treatment with tofacitinib resulted in a substantial increase in the number of hair follicles, with a considerable proportion observed in the active growth phase (Figure 6A). Furthermore, immunofluorescence staining revealed a marked increase in CD8^+^ T cell infiltration in the AA group relative to the Control group, whereas tofacitinib treatment significantly reduced CD8^+^ T cell infiltration (Figure 6B).

## 4 Discussion

Alopecia areata (AA) is an immune-mediated hair loss disorder, typically presenting as patchy areas of hair loss on the scalp, but it can affect anywhere on the body (Gaurav et al., 2024). The loss of immune privilege in hair follicles is a critical factor driving the progression of AA. The condition is influenced by a combination of genetic, environmental, and psychological factors, which leads to the exposure of self-antigens from hair follicle epithelial cells during the anagen phase. This antigen exposure, combined with cytotoxic T-lymphocyte activity and peri-follicular immune responses, disrupts the hair follicle growth cycle, ultimately resulting in hair loss (Sterkens et al., 2021; Yoko et al., 2022). Interferon-γ (IFNγ) has been identified as key cytokines in AA pathogenesis (Markus and Marta, 2020). In a spontaneous mouse model for AA, interferon-gamma-deficient mice are resistant to the development of AA (Pia et al., 2006). Additionally, AA has been recognized as a potential side effect of PD-L1 (programmed death ligand 1) inhibitors and anti-CTLA-4 (cytotoxic T-lymphocyte-associated protein-4) agents, with a reported prevalence of 1.0%–1.06% (Samer et al., 2006; Suzanne et al., 2012; Hazem and Kenneth, 2013; Lars et al., 2016). Studies in spontaneous mouse models of AA have shown that IFN-γ-deficient mice are resistant to the development of the condition. Additionally, AA has been recognized as a potential side effect of PD-L1 (programmed death ligand 1) inhibitors and anti-CTLA-4 (cytotoxic T-lymphocyte-associated protein-4) agents, with a reported prevalence of 1.0%–1.06%.

Other immunological mechanisms involved in AA include decreased expression of immunosuppressive molecules such as transforming growth factor-β (TGF-β), vasoactive intestinal peptide (VIP), and macrophage migration inhibitory factor (MIF), alongside immune responses such as mast cell degranulation (Soraya et al., 2017; Feng et al., 2023).

In addition to immune system dysregulation, AA may also be related to metabolic factors. Metabolic abnormalities are frequently observed in patients with AA, with a notably high incidence of associated metabolic disorders (Rosalynn et al., 2020). Specifically, patients with AA exhibit elevated serum insulin levels, accompanied by decreased while adiponectin and resistin levels (Ayşe Serap et al., 2012; Mohammad et al., 2019; Anna et al., 2021). In our study, using GC-MS-based non-targeted metabolomics analysis, we identified several differential metabolites in AA-affected mice, including D-Lactic acid, glycolic acid, linoleic acid, petroselinic acid and, stearic acid. Among these, Linoleic acid (LA) is an ω-6 polyunsaturated fatty acid that has long been associated with inflammation. In the human body, linoleic acid is converted into arachidonic acid, which integrates into the colonic epithelial cell membrane. Once released from the membrane, arachidonic acid is metabolized into pro-inflammatory eicosanoids, including prostaglandin E2, leukotriene B4, and thromboxane A2 (Yaqoob et al., 2000; Hanna and Hafez, 2018; Wang et al., 2021). These pro-inflammatory eicosanoids are found in the colonic mucosa of patients with ulcerative colitis, and high dietary linoleic acid intake is believed to contribute to the onset of ulcerative colitis (Zhang et al., 2024). Regarding antitumor immunity, studies have shown that linoleic acid serves as a key positive regulator of CD8^+^ T cell activity. It enhances T cell metabolic fitness and promotes their differentiation into memory-like phenotypes with superior effector functions, thereby playing a crucial role in antitumor immunity (Nava Lauson et al., 2023). Additionally, LA is also metabolized into hydroxyoctadecadienoic acids (HODEs) and other derivatives, which further contribute to inflammatory processes (Vangaveti et al., 2016). HODEs, as critical biomarkers of skin lipid peroxidation and oxidation, are elevated in the hair and scalp of patients with seborrheic dermatitis (SD) (Hordinsky et al., 2025). In our study, non-targeted metabolomics analysis using GC-MS identified linoleic acid as a differential metabolite in the serum of control and alopecia areata mice. Following tofacitinib treatment, serum linoleic acid levels decreased. These findings suggest that linoleic acid metabolic dysregulation may be a potential contributor to the development of alopecia areata in mice.

Trace elements such as zinc and selenium also play a crucial role in the functional activities of hair follicles. A decrease in zinc and levels, coupled with an increase in cadmium, cobalt, and lead in the human body, may promote the pathogenesis and development of AA (Xiran et al., 2019). In this study, we identified four differential ions—magnesium, aluminum, titanium, and nickel—in the serum of mice with AA using an ICP-MS-based ionomics approach for the first time. Magnesium (Mg) is an essential micronutrient in mammalian cells, serving as a critical cofactor for hundreds of enzymes. Increasing evidence highlights the significant role of metal ions in regulating various aspects of the human immune response. Magnesium deficiency has been closely associated with the development of several diseases, including infections and cancer (Larsson et al., 2012; Costello and Nielsen, 2017). Currently, studies have shown that Mg^2+^ influence immune system-related phenotypes and their underlying mechanisms. For instance, in 2011, Li et al. found that in patients with a deficiency of the Mg^2+^ transporter MAGT1, T cells failed to activate due to the inability of Mg^2+^ to enter the cells, thereby disrupting downstream PLCγ-mediated signaling (Li et al., 2011). In 2013, they further discovered that Mg^2+^ regulate the function of NK and CD8^+^ T cells through NKG2D. Compared to healthy controls, patients with MAGT1 deficiency exhibited significantly reduced NKG2D expression on NK and CD8^+^ T cells, along with a markedly increased serum EBV viral load, highlighting the crucial role of Mg^2+^ in antiviral immunity (Chaigne-Delalande et al., 2013). Additionally, research by Jonas Lötscher et al. demonstrated that CD8^+^ T cells can efficiently eliminate abnormal or infected cells only in a magnesium-rich environment. Further investigations revealed that Mg^2+^ are essential for the function of the co-stimulatory molecule LFA-1 on the surface of CD8^+^ T cells. Moreover, Mg^2+^ play a pivotal role in infection immunity, CAR-T therapy, and immune checkpoint therapy (Lötscher et al., 2022). These findings further underscore the indispensable role of Mg^2+^ in CD8^+^ T cell activation and function.

Alopecia areata is a common autoimmune disease closely associated with the disruption of the immune privilege mechanism of hair follicles. CD8^+^ T cells are key players in the pathogenesis of alopecia areata, attacking hair follicles in conjunction with a significant IFN-γ response and upregulation of γ-chain cytokines, which subsequently activate the JAK-STAT pathway, triggering inflammation and follicular damage (Guo et al., 2024). Furthermore, the expression of MHC class I chain-related gene A (*MICA*), a stress-induced ligand, is notably elevated in hair follicles affected by alopecia areata, coinciding with increased CD8^+^ T cell infiltration (Fehrholz and Bertolini, 2020). In our study, using ICP-MS-based ionomics analysis, we identified Mg^2+^ as key differential ions in the serum of control and alopecia areata mice. Treatment with tofacitinib reduced serum magnesium ion levels and significantly decreased CD8^+^ T cell infiltration. These findings suggest that Mg^2+^ may contribute to alopecia areata by activating CD8^+^ T cells and enhancing their function, thereby promoting disease progression.

In this study, We established an AA mouse model using KM mice through topical application of sodium sulfide (Na_2_S), followed by tofacitinib administration to investigate its therapeutic efficacy. The selection of Na_2_S as a chemical irritant was based on its capacity to simulate inflammatory responses in the scalp microenvironment. Post-inflammatory infiltration of CD8^+^T cells in perifollicular regions demonstrated pathophysiological parallels with human AA progression, thereby facilitating efficient evaluation of drug efficacy. However, there are still some limitations in this study. Compared with C3H/HeJ mice, KM mice are not capable of producing spontaneous AA and therefore cannot mimic the immune-mediated nature of AA, which is a limitation in the in-depth investigation of the pathogenesis of AA. To address this constraint, our subsequent investigations will employ C3H/HeJ mice combined with imiquimod-induced modeling. This refined approach will enable systematic exploration of AA pathogenesis across disease progression stages, precise delineation of molecular mechanisms, and discovery of novel therapeutic targets. Limitations in other areas also suggest directions for future research, such as: differences between species in hair follicle cycles and immune regulation may affect the translational relevance of the findings. The hair cycle in mice is synchronous, whereas human hair follicles operate asynchronously, which could alter the interactions between immune cells during the development of AA. Additionally, although the Mg^2+^ levels in mice (1.38 ± 0.12 mM) are similar to the physiological range in humans (0.75–1.02 mM), dietary heterogeneity may influence the correlation of ion metabolites. Moreover, differences in metabolic pathways between species may impact the representativeness of the experimental results. To address these limitations, our future studies will consider human-centered validation strategies. Longitudinal analysis of scalp biopsies from AA patients using single-cell RNA sequencing could resolve the issue of immune cell heterogeneity and identify human-specific therapeutic targets. Furthermore, clinical trials can validate candidate biomarkers and therapies in different patient cohorts. Additionally, integrating patient-derived autoreactive CD8^+^ T cells with organoid-based models may help bridge the translational gap.

In conclusion, Our study represents the first comprehensive metabolomics-ionomics conjoint analysis of alopecia areata samples, offering novel insights and potential research directions for the prevention and treatment of this condition. This research highlights the multifaceted nature of AA and underscores the need for a more integrated understanding of its etiology to develop effective therapeutic strategies.

## Data Availability

The original contributions presented in the study are included in the supplemental material, and further inquiries can be directed to the corresponding author.

## Abbreviations

AA, Alopecia areata; IL-2, interleukin-2; IL-15, interleukin-15; GC-MS, gas chromatography-mass spectrometry; ICP-MS, inductively coupled plasma mass spectrometry; ROC, receiver operating characteristic curve; KEGG, Kyoto Encyclopedia of Genes and Genomes; FMT, fecal microbiota transplantation; OPLS-DA, orthogonal partial least squares-discriminant analysis; IFNγ, Interferon-γ; PD-L1, programmed death ligand 1; TGF-β, transforming growth factor-β; VIP, vasoactive intestinal peptide; MIF, macrophage migration inhibitory factor; LA, linoleic acid; HODEs, hydroxyoctadecadienoic acids; ARA, arachidonic acid; *MICA*, MHC class I chain-related gene A.
