# Supplementary figures and images for: Associations between ionomic profile and metabolic abnormalities in a murine model of sodium sulfide induced alopecia areata
Source: Front Pharmacol. 2025 May 14;16:1507348. doi: 10.3389/fphar.2025.1507348 (PMC12117264; doi:10.3389/fphar.2025.1507348)

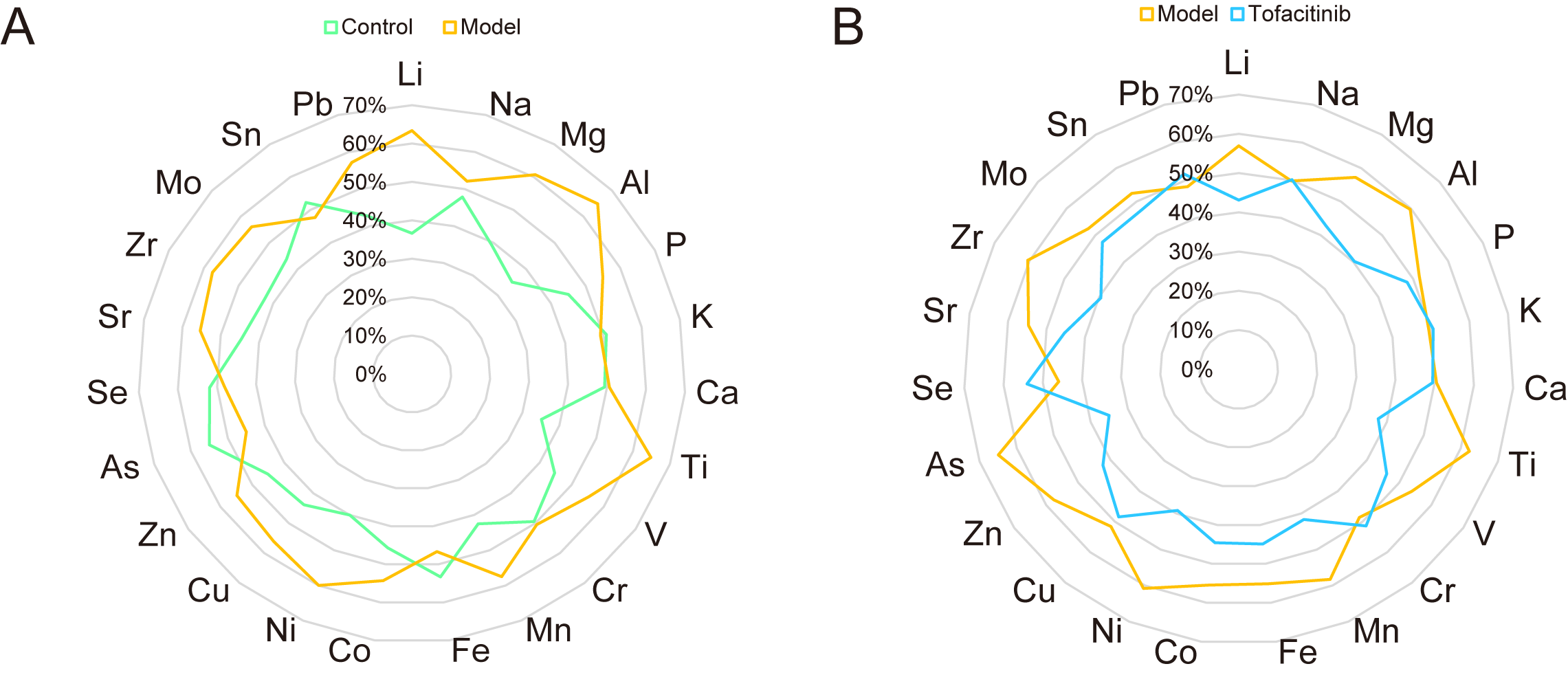

Supplement: Supplementary file 1 [file Image3.tif]

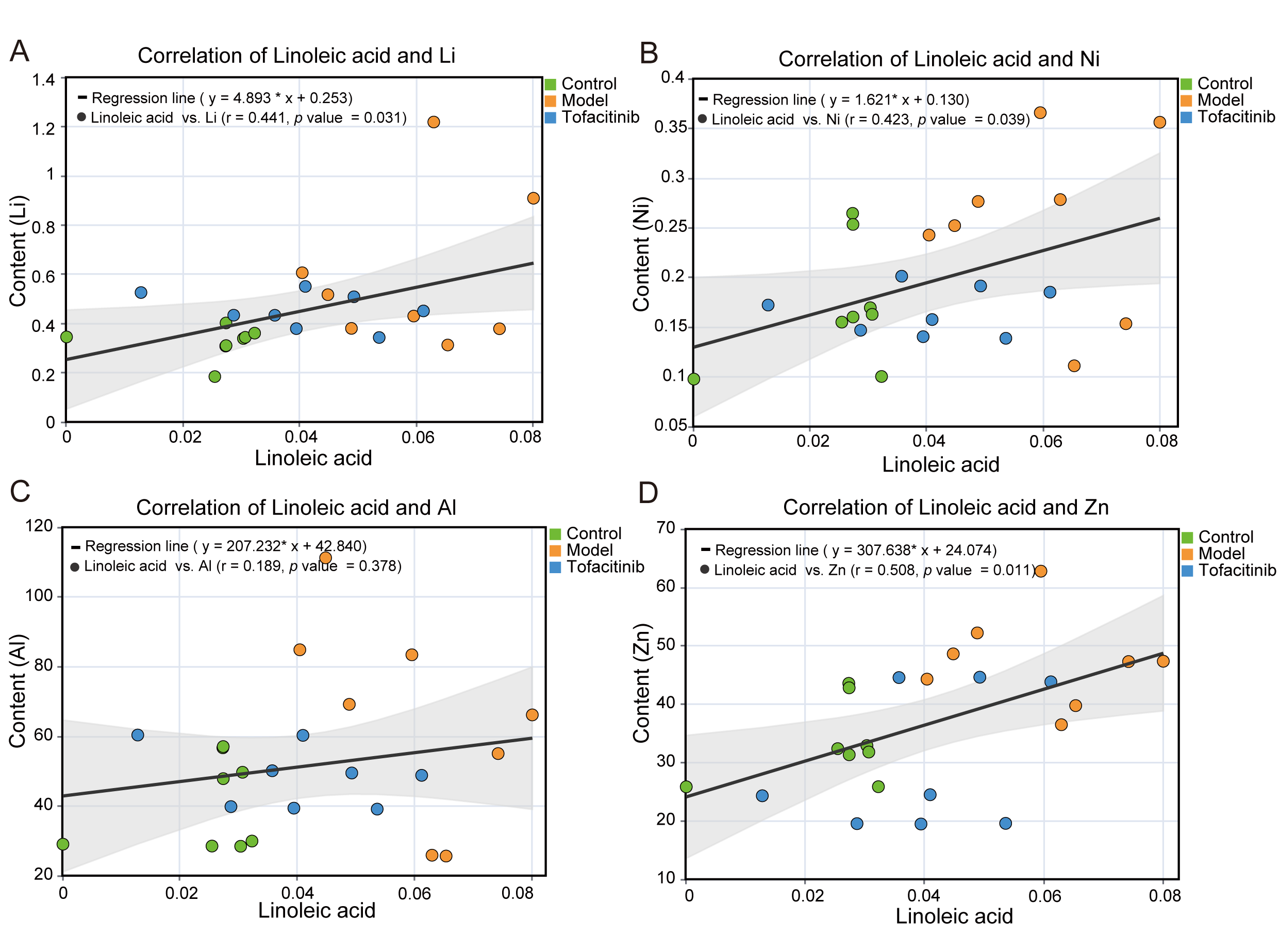

Supplement: Supplementary file 2 [file Image4.tif]

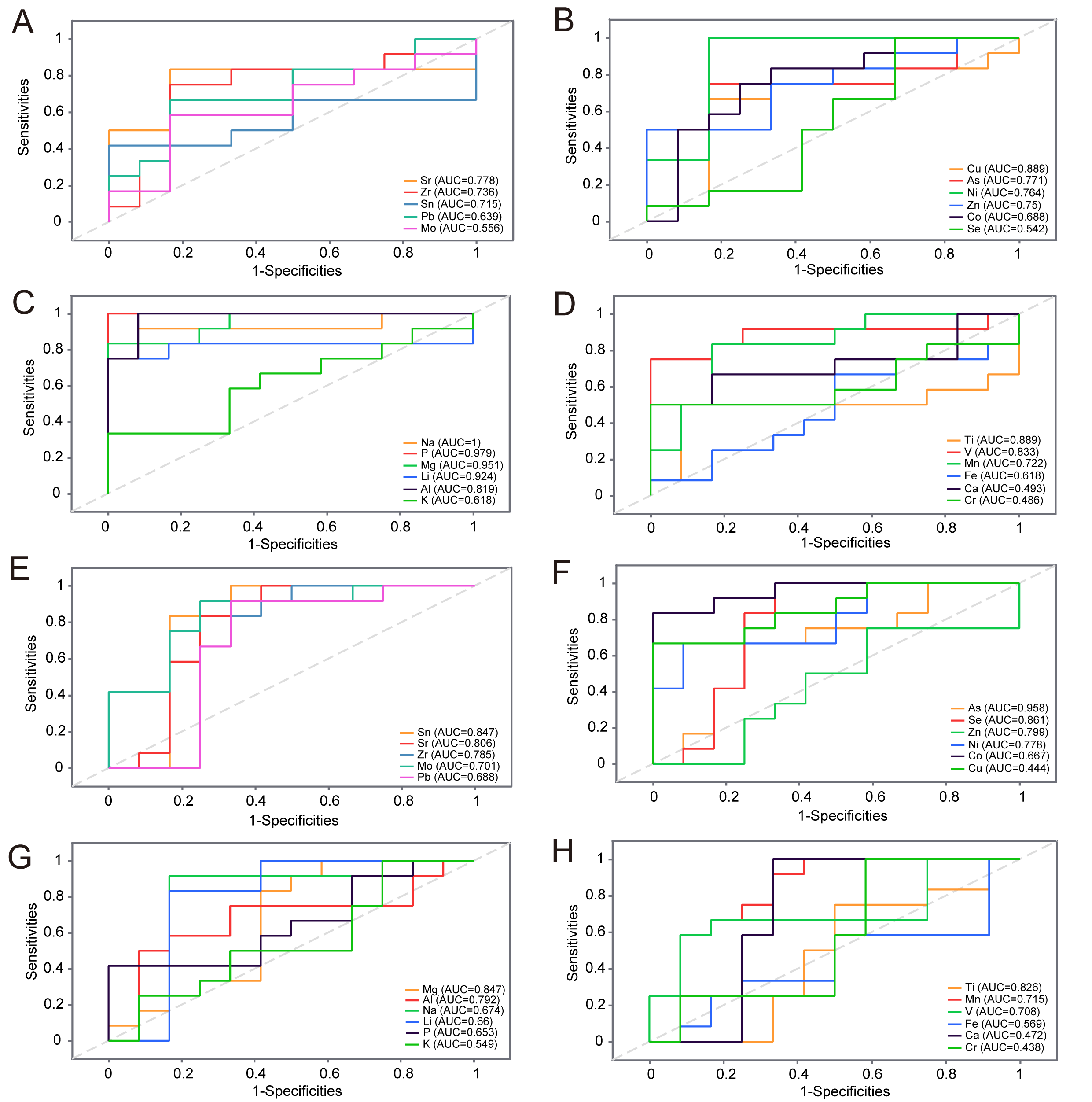

Supplement: Supplementary file 3 [file Image2.tif]

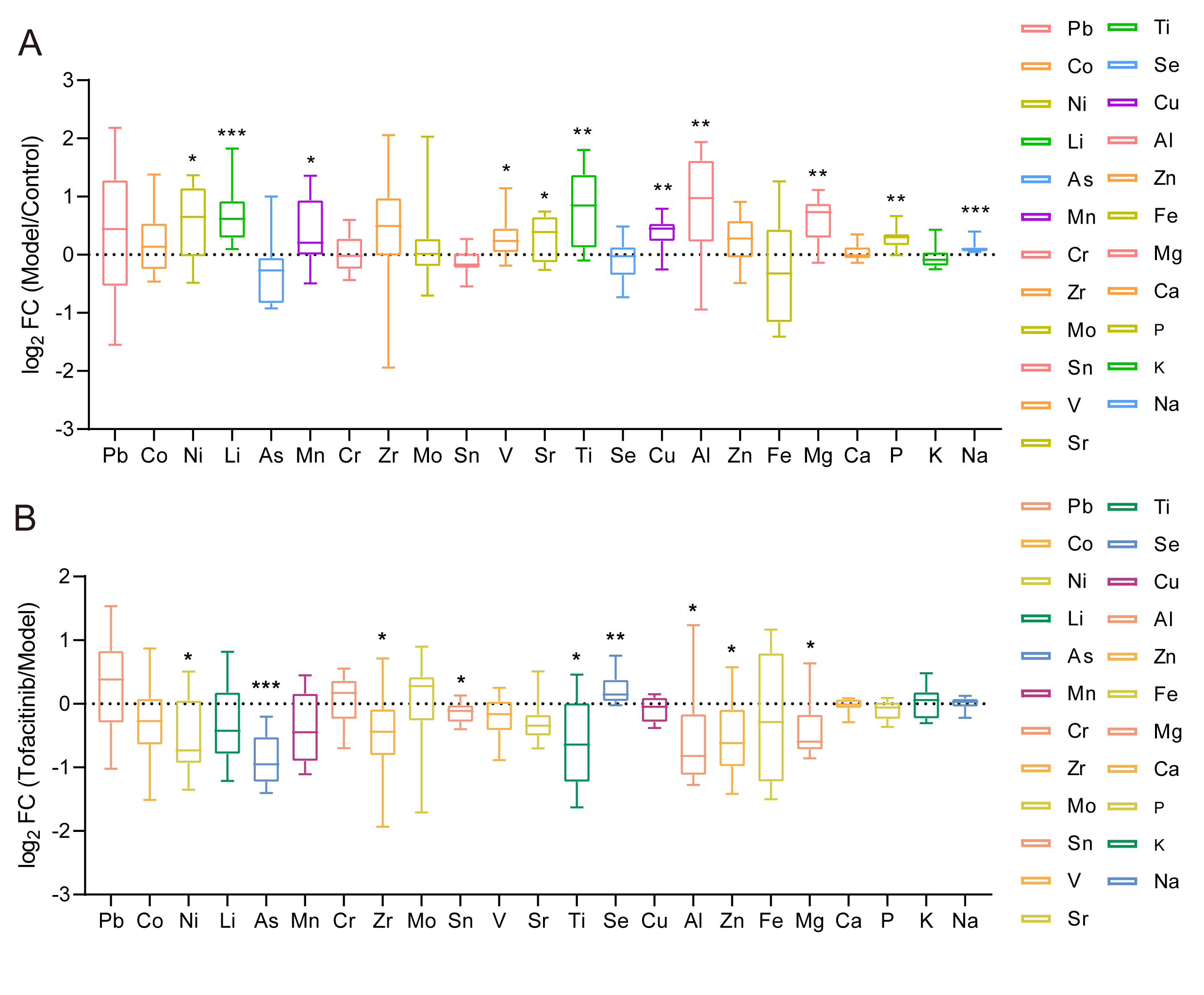

Supplement: Supplementary file 4 [file Image1.tif]
